# Fever of unknown origin and splenomegaly: A case report of blood culture negative endocarditis

**DOI:** 10.1097/MD.0000000000009197

**Published:** 2017-12-15

**Authors:** Maria Livia Burzo, Mariangela Antonelli, Giovanni Pecorini, Angela M.R. Favuzzi, Raffaele Landolfi, Andrea Flex

**Affiliations:** a Institute of Internal Medicine; b Laboratory of Vascular Biology and Genetics, Catholic University of the Sacred Heart, Fondazione University Hospital A. Gemelli, Rome, Italy.

**Keywords:** echocardiogram, endocarditis, fever, splenomegaly, valvular disease

## Abstract

**Rationale:**

Fever of unknown origin (FUO) can be determined by different conditions among which infectious diseases represent the main cause.

**Patient concerns:**

A young woman, with a history of aortic stenosis, was admitted to our unit for a month of intermittent fever associated with a new diastolic heart murmur and splenomegaly. Laboratory tests were negative for infectious screening. The total body computed tomography (CT) scan excluded abscesses, occulted neoplasia, or lymphadenopathy.

**Diagnoses:**

The transthoracic and transesophageal echocardiogram showed an aortic valve vegetation. Three sets of blood cultures were negative for all microorganisms tested. According to these findings, Bartonella endocarditis was suspected and the serology tests performed were positive. Finally, real-time polymerase chain reaction (RT-PCR) detected *Bartonella henselae* DNA on tissue valve.

**Interventions:**

The patient underwent heart valve surgery and a treatment of Ampicillin, Gentamicin, and oral Doxycycline was prescribed for 16 days and, successively, with Doxycycline and Ceftriaxone for 6 weeks.

**Outcomes:**

After surgery and antibiotic therapy, patient continued to do well.

**Lessons:**

Bartonella species are frequently the cause of negative blood culture endocarditis. Molecular biology techniques are the only useful tool for diagnosis. Valvular replacement is often necessary and antibiotic regimen with Gentamicin and either Ceftriaxone or Doxycycline is suggested as treatment.

Echocardiogram and blood cultures must be performed in all cases of FUO. When blood cultures are negative and echocardiographic tools are indicative, early use of Bartonella serology is recommended.

## 1 Introduction

Fever of unknown origin (FUO) was defined in 1961 by Petersdorf and Beeson as the following: a temperature greater than 38.3°C (101°F) on several occasions, more than 3 weeks duration of illness, and failure to reach a diagnosis despite 1 week of inpatient investigation. The causes generally recognized are infections, rheumatic disease, malignancy or other (factitia, sarcoidosis, drugs). Infections and noninfectious inflammatory diseases each account for 15% to 35% of FUOs, while malignancies cause less than 20% of these fevers.^[1,2]^ In the current case series, cases of FUO that remain undiagnosed account from 3% to 25%.^[2,3]^

Infective endocarditis (IE) represent one of the most common causes of FUO. Even if clinical manifestations of IE are highly variable, fever is the most common symptom of IE (up to 90% of cases). It is often associated with chills, anorexia and weight loss, malaise, headache, myalgias, and arthralgias. Cardiac murmurs are observed in approximately 85% of patients and supportive signs include splenomegaly and cutaneous manifestations, such as petechiae or splinter hemorrhages.

The diagnosis of IE should be suspected in all patients with fever (with or without bacteremia) in the setting of relevant cardiac (previous endocarditis, intracardiac devices, pre-existing valve damage) and noncardiac (such as occupational history, animal contacts, immunization status) risk factors. The diagnosis is based on clinical manifestations, blood cultures (or other microbiologic data), and echocardiography (transthoracic or transesophageal), according to the accepted modified Duke criteria.^[4]^

Typical microorganisms consistent with IE include Staphylococcus aureus, viridans streptococci, community-acquired enterococci, *Streptococcus gallolyticus*, or HACEK (Haemophilus, Aggregatibacter, Cardiobacterium, Eikenella, Kingella) organisms.

Blood culture negative endocarditis (BCNE) is defined as endocarditis without etiology following inoculation of at least 3 independent blood samples in a standard blood-culture system, with negative cultures after 5 days of incubation and subculturing and may account for up to 30% of all IE.

Blood cultures may remain negative from 2% up to 7% of cases, even when the utmost care is taken in obtaining the proper number and volume of blood cultures.^[5]^ It can be possible for 3 major reasons: previous administration of antimicrobial agents, inadequate microbiological techniques, or infection with highly fastidious bacteria or nonbacterial pathogens. Some of pathogens that can cause BCNE are Bartonella species, which are fastidious Gram-negative bacilli not growing unless special media or microbiologic methods are used.

Of the 24 species including the Bartonella genus, *Bartonelaa bacilliformis*, *Bartonella henselae*, and *Bartonella quintana* infections are most commonly implicated in human disease, and *B. henselae* and *B. quintana* account for the majority of morbidity and mortality among immunocompromised individuals. They have an identified zoonotic reservoir, in particular, *B. henselae* is a zoonosis transmitted from natural reservoir, cats, usually via cat scratch or bite and less commonly by a vector such as cat fleas or ticks.

## 2 Case report

A 54 -year-old woman was admitted to our Department for an intermittent fever lasted from 1 month, with shivering and temperature greater than 39.0°C, weight loss, and fatigue.

The patient had a history of aortic stenosis symptomatic for dyspnea and exertional angina, diagnosed 1 year before, for which she was evaluated for cardiac elective surgery. The patient lived and worked on a farm and had continuous contact with stray animals, including a cat.

Upon admission, she was afebrile and physical examination revealed a new diastolic heart murmur and splenomegaly.

Routine laboratory tests performed were normal, except for the evidence of pancytopenia, polyclonal gammopathy, and a slight rise of transaminases. C-reactive protein (CRP) was 4 mg/dL (normal value <0.5 mg/dL), and procalcitonin was 0.30 ng/mL (normal value <2.0 ng/mL).

Serology for hepatitis B and C virus, Leptospira, *Toxoplasma Gondii*, Cytomegalovirus, Epstein–Barr virus (EBV), and herpes simplex virus (HSV) were tested and acute infections were excluded. Finally, serology for HIV infection and Quantiferon test resulted negative.

**Figure 1:**
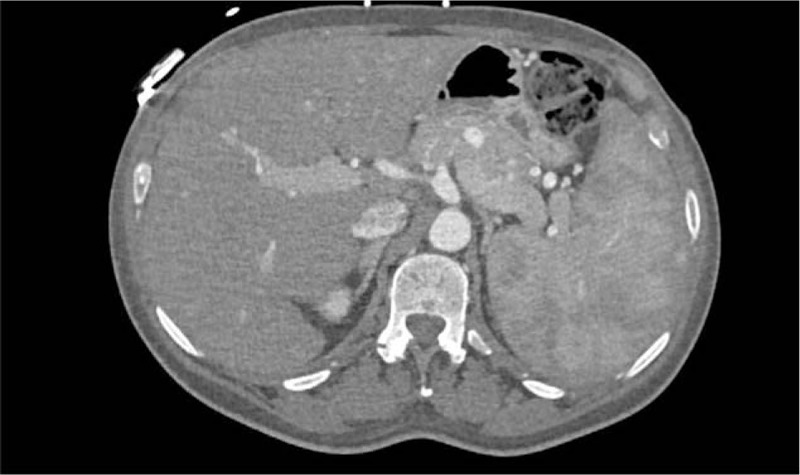
Severe splenomegaly at CT abdomen scan.

**Figure 2:**
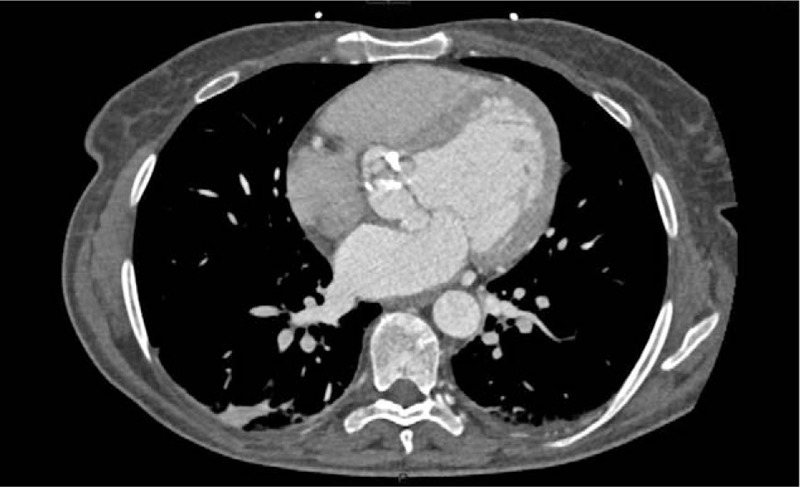
Extended aortic valve calcification at CT chest scan.

A brain–chest–abdomen CT scan showed severe aortic valve's stenosis with calcifications and confirmed the presence of severe splenomegaly, however, not documenting any occulted neoplasm, lymphadenopathy, or abscesses (Figs. 1 and 2).

According to ESC Guidelines,^[4]^ a transthoracic echocardiogram was performed in order to exclude IE. It showed an aortic valve diffusely echogenic and altered as for endocarditis, with Doppler evidence of moderate stenosis and severe insufficiency (aortic transvalvular gradient of 27 mm Hg) with multiple and variously oriented jets, due to flat valve overhanging. Moreover, aortic valve ring was disomogeneously thickened as for a perivalvular abscess and enlargement of left Valsalva sinus.

Thus, a transesophageal echocardiogram was performed in order to evaluate the perivalvular abscesses. It showed a bicuspid aortic valve with small floating training in the outflow tract (5 mm) and confirmed the left Valsalva sinus's aneurysm (the maximum size of 21 × 11 mm; 1.34 cm^2^ area) with dilatation of right Valsalva sinus too, findings compatible with abscessualized aortic valve endocarditis.

Three sets of blood cultures collected by BACTEC bottles were obtained 6 hours away from each other and from separate sites.

Then, empiric antimicrobial drug therapy, including Gentamycin and Ampicillin, was initiated.

After 5 days of incubation in fresh agar plates maintained in 5% CO_2_ at 35°C, all blood culture samples resulted negative for bacterial growth, and no direct microscopy nor subculture of blood samples were made, as protocol of our Institute's microbiology laboratory.

As indicated by ESC Guidelines in case of BCNE,^[4]^*Coxiella burnetii*, *Legionella pneumophila*, *Clamydia pneumoniae*, Brucella, Aspergillus, and Bartonella species serology were tested. Only immunoglobulin G and M for *B. henselae* were positive, with a titer of 1/2560 and 1/800, respectively. Then, oral Doxycycline was prescribed for the treatment of presumed Bartonella endocarditis.

Given the echocardiographic finding of extensive remodeling of aortic valve, with fluctuating vegetation, 2 days later, the patient underwent aortic valve and aortic root replacement due to the high risk of septic embolization.

After surgery, patient received a treatment with Ampicillin, Gentamicin, and Doxycycline for 16 days and, successively, with Doxycycline and Ceftriaxone for 6 weeks.

Gram staining, acid-fast staining, and fungal staining of patient's aortic valve cups showed that no microorganisms were present in valvular tissues. Culture analyses of the vegetation and cardiac valvular tissue samples by recommended techniques^[6]^ were negative for any bacterial and fungal species after 28 days of incubation.

Finally, RT-PCR detected *B. henselae* DNA on tissue valve.

The ethical committee approval was not necessary. Informed consent was not obtained because the patient was discharged long before we wrote the case report.

## 3 Discussion

Bartonella endocarditis accounts for 5% to 30% of all endocarditis cases.^[5]^ To date, almost all reports have involved adults and, overall, approximately 85% to 90% of the cases have involved men.

Available data suggest that homelessness, alcoholism, and infestation with body lice are associated with *B. quintana* endocarditis, whereas contact with cats and previous valvular disease serve as the major risk factors for *B. henselae* endocarditis,^[7]^ as did our patient.

The clinical features are similar to those of patients having other IE, as described by the Duke Criteria.^[4]^ Affected patients typically present with subacute, nonspecific symptoms, including fever, fatigue, weakness, and weight loss. In addition, most patients have evidence of a murmur on cardiac auscultation.

Such patients typically have an aggressive disease course characterized by valvular perforation or rapid valvular damage progression and heart failure. Embolic events have been reported in some case series, even if available data are inconsistent.^[8]^

Approximately 90% of patients have involvement of the aortic valve and, approximately, in 50% of them, there is the evidence of a preexisting valvular disease.^[8]^

Our patient presented to us with 1-month-old intermittent fever with shivering, weight loss, fatigue, and physical examination revealed a new diastolic murmur and splenomegaly.

Both laboratory tests and chest-abdomen CT scan did not reveal any clues for diagnosis. Echocardiographic findings were strongly suspected for endocarditis, but all the 3 sets of blood cultures performed were negative after 72 to 96 hours of incubation.

As indicated by ESC guidelines,^[4]^ serology for Bartonella infection was performed and resulted positive. Finally, diagnosis was confirmed by the reply of *B. henselae* DNA on tissue valve, after elective surgery.

To date, there is still a lack of criteria for diagnosis of Bartonella endocarditis. Blood cultures, in fact, are often negative and the serologic methods currently used are limited by their poor specificity (in some case series, a cross-reaction between Bartonella species and Coxiella and Clamydia species was demonstrated) and by the absence of a defined significant titers, even if an IgG titer of >800 could be considered significant.^[9]^

Among molecular biology techniques, RT-PCR represents a helpful method for demonstrating *B. henselae* infection, by detecting bacterial DNA in cardiac valvular tissue, especially when fresh valvular tissue is used. Furthermore, PCR can distinguish all of Bartonella species, unlike serological tests.

At present, the optimal antibiotic therapy for Bartonella endocarditis is unknown, as there are no randomized trials. In addition, many patients with Bartonella endocarditis have undergone cardiac valvular surgery with removal of the infected valve, making it difficult to assess the separate role of antibiotics.

However, treatment guidelines for suspected and proven Bartonella endocarditis were defined in the 2005 American Heart Association (AHA) Infective Endocarditis guidelines.^[10]^ Initial regimen for suspected Bartonella endocarditis should include Ceftriaxone, Doxycycline, and Gentamicin. After Bartonella endocarditis was proven, treatment suggested consists of the use of Doxycycline and Gentamicin for 14 days. After that, patients should receive an extended course of Doxycycline monotherapy. The duration of Doxycycline monotherapy depends upon whether or not cardiac valvular surgery was performed to remove the infected valve: 6 weeks for patients undergone cardiac valve surgery and 3 months for those not undergoing surgery.

## 4 Conclusion

We diagnosed a native valve endocarditis caused by *B. henselae* in a peasant woman by using serology and PCR analysis of surgical tissue samples. The patient underwent aortic valve replacement and then received a course of Doxycycline and Ceftriaxone, continuing to do well.

In conclusion, transthoracic echocardiogram and blood cultures remain the cornerstone of diagnosis of IE and they must be performed in all cases of FUO.

Bartonella infection should be included in the differential diagnosis of culture-negative endocarditis. Early use of Bartonella serology (as well as Coxiella burnetii, Chlamydia, Legionella, Aspergillus, and Brucella serology) is recommended in those cases in which properly obtained blood cultures do not establish the etiology of the endocarditis. Nonculture techniques such as serology and PCR analysis of surgical tissue may be the diagnostic methods of choice given the difficulty in culturing these organisms.
